# Communicating cancer risk in the primary care consultation when using a cancer risk assessment tool: Qualitative study with service users and practitioners

**DOI:** 10.1111/hex.13016

**Published:** 2020-01-22

**Authors:** Joseph N. A. Akanuwe, Sharon Black, Sara Owen, Aloysius Niroshan Siriwardena

**Affiliations:** ^1^ Community and Health Research Unit School of Health and Social Care University of Lincoln Lincoln UK; ^2^ Waterford Institute of Technology Waterford Ireland

**Keywords:** cancer risk assessment tools, cancer risk communication, general practitioners, primary care, QCancer, service user views

## Abstract

**Background:**

Cancer risk assessment tools are designed to help detect cancer risk in symptomatic individuals presenting to primary care. An early detection of cancer risk could mean early referral for investigations, diagnosis and treatment, helping to address late diagnosis of cancer. It is not clear how best cancer risk may be communicated to patients when using a cancer risk assessment tool to assess their risk of developing cancer.

**Objective:**

We aimed to explore the perspectives of service users and primary care practitioners on communicating cancer risk information to patients, when using QCancer, a cancer risk assessment tool.

**Design:**

A qualitative study involving the use of individual interviews and focus groups.

**Setting and participants:**

Conducted in primary care settings in Lincolnshire with a convenience sample of 36 participants (19 service users who were members of the public) and 17 primary care practitioners (general practitioners and practice nurses).

**Results:**

Participants suggested ways to improve communication of cancer risk information: personalizing risk information; involving patients in use of the tool; sharing risk information openly; and providing sufficient time when using the tool during consultations.

**Conclusion:**

Communication of cancer risk information is complex and difficult. We identified strategies for improving communication with patients involving cancer risk estimations in primary care consultations.

## 1 BACKGROUND

Delayed diagnosis of cancer can adversely affect treatment options, outcomes such as survival and quality of life, and costs.^1^, ^2^, ^3^ To tackle late diagnosis of cancer, there has been an increased interest in the use of cancer risk assessment tools to identify and quantify cancer risk in symptomatic individuals during primary care consultations.^4^, ^5^, ^6^, ^7^

Two cancer risk assessment tools designed for symptomatic individuals and available in primary care are QCancer^5^, ^6^, ^7^ and the Risk Assessment Tool [RAT].^4^ Both QCancer and RAT were developed, tested,^4^, ^5^ and QCancer in particular has been independently validated,^8^, ^9^ as accurately quantifying risk of cancer in primary care. Despite this, it is currently not clear how cancer risk information generated through use of the tools can be effectively communicated to patients.

Current evidence suggests that discussion of risk information was less likely to occur if patients did not prompt practitioners.^10^ In a recent review of the literature around cancer risk assessment tools in primary care, a key challenge identified was how best to communicate risk information to patients^11^ without causing undue anxiety or worry to patients.

Exploring ways to best communicate cancer risk to patients is important as the NHS cancer plan advocates effective communication between health professionals and patients, which can facilitate the delivery of high‐quality care and empower people to be involved in decisions about their care.^12^ While there is existing literature about communicating and sharing decisions with people who have cancer,^13^ most communication evidence relates to people with existing cancer, but is not directly related to the use of cancer risk assessment tools designed for individuals with symptoms reporting to primary care who may not yet be aware of their cancer status. In fact, recent research in this area indicates cancer decision support tools which include QCancer are not being widely used.^14^

The aim of this study was to explore the views of service users and primary care practitioners on how best to communicate cancer risk information when using QCancer, a cancer risk assessment tool, with symptomatic individuals in primary care consultations to enable them be involved in decisions on referral and cancer investigations. QCancer was used as a reference tool because it has been validated externally.^8^, ^9^

## 2 METHODS

### 2.1 Design and setting

We used a qualitative design employing semi‐structured individual interviews and focus groups. The study took place in Lincolnshire in the East Midlands region of England during 2016. Ethical approval for the study was granted by the School of Health and Social Care Ethics Committee, University of Lincoln.

The eight stages of the Risk Analysis Framework^15^ informed the development of the topic guide for the interviews, the analysis and interpretation of the data as well as the discussion of the findings. The framework recognizes that a combination of some or all of the stages is important for effectively communicating risk to patients.^15^ The eight stages are (to some extent ironically) stated as follows: (a) Get the numbers right; (b) Tell them the numbers; (c) Explain what the numbers mean; (d) Show them that they have accepted similar risks in the past; (e) Show them that it is a good deal for them; (f) Treat them nicely; (g) Make them partners; and (h) Combine all the stages. The Risk Analysis Framework was selected to inform the development of the topic guides (for the interviews and focus groups), the analysis and interpretation of the data, because the various stages of the framework served as a suitable theoretical background for explaining how best cancer risk can be communicated to a patient during consultations.

### 2.2 Recruitment of participants and data collection

We recruited a convenience sample of service users and primary care practitioners (GPs and practice nurses) for the study. Table 1 presents the characteristics of participants with regard to age group, gender and ethnicity. Service users were recruited using flyers placed at public places including community centres and libraries in the study area and through a patient and public involvement group.

**Table 1:** Participant characteristics

	Service users	Practitioners
Gender		
Male	7	13
Female	12	4
Age group		
20‐29	3	—
30‐39	4	3
40‐49	1	10
50‐59	3	4
60‐69	5	—
70‐79	3	—
Ethnicity		
White British	19	6
Indian	—	6
Pakistani	—	3
Asian British	—	1
Bangladeshi	—	1
Practice patient list size		
200‐2900	—	1
3000‐3900	—	—
4000‐4900	—	—
5000‐5900	—	—
6000‐6900	—	8
7000‐7900	—	—
8000‐8900	—	—
9000‐9900	—	8

Our sample of service users included adults who did not have active cancer but could potentially present to general practice with symptoms suggesting cancer, where a clinician might use the tool during the consultation. Patients with known cancer were not included because the cancer risk assessment tool of interest is designed to help detect risk of cancer in symptomatic individuals and would not be used in the presence of known cancer. Although patients with cancer could have provided useful views, we did not include them because of the potential stress of taking part. Similarly, our sample of practitioners included GPs and practice nurses who usually see patients who present to general practice with symptoms which could be those of cancer. We checked with service users and confirmed that they were adults and had no active cancer at the time of being interviewed. We also confirmed with practitioners that they worked in general practices in the study area and were involved in patient consultations, particularly for practice nurses.

Interested service users contacted the researcher (JNA) for more information and to arrange a suitable date and time for individual interviews. Interviews were conducted either in service users’ own homes or at the university. Individual interviews were used for service users as it was not possible to meet them in groups.

Primary care practitioners were invited to participate through local general practices. Interested practitioners contacted the researcher for more information, and to arrange a date and time for either individual interviews or focus groups. Practitioners whose schedules did not allow them to meet in groups were offered individual interviews instead. Groups of practitioners in practices who agreed to be interviewed together were offered a focus group.

**Figure 1:**
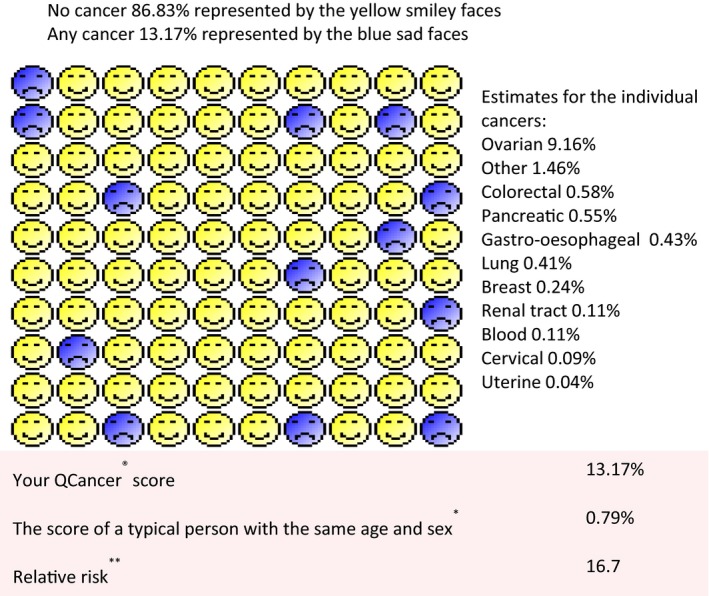
Vignette (example) of QCancer risk scores

At the beginning of interviews and focus groups with participants, a vignette of how the QCancer tool (the reference tool for this study) works was shown to participants. Where there was Internet access, the researcher (JNA) demonstrated how QCancer worked. Where Internet access was unavailable, the vignette was explained in terms of how an individual's risk factors and symptoms could be entered in the QCancer tool to calculate a cancer risk represented by blue sad faces (those at risk of having cancer within two years) and yellow smiley faces (those not at risk of cancer) as shown in Figure 1. After showing and explaining or demonstrating the vignette to participants, questions were asked to explore views on how best cancer risk information could be communicated to patients.

Prior to starting the individual interviews and focus groups, participants were asked to give written consent and were assured that they were free to discontinue the interview or focus group at any point. With permission of the participants, the interviews and focus groups were audio recorded and later transcribed verbatim. Notes were also taken which complemented the audio‐recorded data.

### 2.3 Data analysis

Data were transcribed verbatim and analysed using the framework approach^16^ facilitated by NVivo version 10. A priori codes derived from the Risk Analysis Framework informed the interview guide, analysis and interpretation by providing an initial coding framework of deductive themes arising from the relevant parts of the Risk Analysis Framework such as ‘get the numbers’, ‘tell them the numbers’, ‘explain what the numbers mean’, ‘treat them nicely or with respect’ and ‘make them partners’.

Further inductive codes were identified from the data. This involved the following steps. Two investigators (JNA and ANS) read and re‐read transcripts to derive an initial coding framework which was discussed and agreed by the wider research team. Initial descriptive themes were developed by organizing codes. These were developed iteratively through further interpretation and discussion into a smaller number of overarching themes. For both service users and practitioners, data were collected and analysed until saturation was achieved; that is, no new codes or meaning were identified.^17^ Data from service users and practitioners were analysed separately and then compared to determine the extent to which these views agreed or differed.

### 2.4 Patient and public involvement

The research questions, the study design and interview guides were informed through discussion with the Healthier Aging Patient and Public Involvement (HaPPI) group at the University of Lincoln. Members of the HaPPI group also helped with recruitment of service users, helping to identify and distribute the study flyers to interested participants.

## 3 RESULTS

### 3.1 Participants

We interviewed 36 participants, 19 service users (aged 21 to 71 years) and 17 practitioners (aged 33 to 55 years) as presented in Table 1. Of the 19 service users, two had a previous diagnosis of cancer, and the rest had relatives or friends who had a previous diagnosis of cancer.

### 3.2 Themes

The analysis produced four overarching themes which addressed the research question on how best to communicate cancer risk information to patients when using a cancer risk assessment tool. Participants felt that effective communication of cancer risk information between patients and practitioners when using cancer risk assessment tools was important, and that this could be enhanced in the following ways: personalizing risk information; involving patients when using the tools; being open and honest; and providing time for listening, informing, explaining and reassuring patients in a professional manner.

#### 3.2.1 Theme 1: Personalizing risk information

This theme relates to the risk communication framework concepts of telling patients and explaining to their level of understanding of what the cancer risk information means. It also relates to the notion that seeing a pictorial illustration of a phenomenon like cancer risk makes the explanation less abstract and enhances understanding of the information being presented to the individual. Indeed, participants felt that personalizing the risk information could help individual patients to understand the risk information being communicated to them. For example, using pictures suitable to individual patients to explain risk information to them was felt to make the information less abstract and easier for patients to understand. One service user said:

I really like this. I like the pictorial representation, I like the fact that it is simple but it's effective because it draws you really right to the point. You know the happy smiley and sad faces can get you an idea. I think it is simple and clear so most people will be able to understand this and take that information on board as opposed to if the doctor just mentions cancer risk, it will put you off. But if you look at this and they talk you through this I think that will be really useful. (Service User 12: individual interview)

Practitioners generally agreed with this view that personalizing risk could enhance patients’ understanding of risk information, but some practitioners felt that the icon arrays used in QCancer could be improved, by arranging them together in rows rather than scattered throughout the diagram (see Figure 1). In line with this, one practitioner said:

I like the smiley faces as well, it's a good way of showing things. But I think it should be lined not random. I will like the blue sad faces to be in line, in a row, otherwise if they are scattered it gives the impression that they are many when they are not. And it's easier to read when they are lined in rows. (Practitioner 11 [GP]: Focus Group 2)

#### 3.2.2 Theme 2: Informing and involving patients

This theme relates to the risk communication framework stage of explaining to patients what cancer risk means and suggests that if cancer risk information is not explained, patients are less likely to feel informed or understand the information, which could make them more anxious, less reassured, and they may lose trust in the clinician.

Practitioners explained their approach to informing patients. One stated:

We just discuss something like ‐ I am worried about this, something sinister, it could be cancer and…. we would like to do a referral, urgent referral for you. That's how we try to explain to the patient. For any other risk, like we do for heart disease. (Practitioner 11 [GP]: individual interview)

Service users felt that it would be helpful to see practitioners using the tools, allowing them to see the information displayed on the computer screen which would help practitioners to explain, and enhance their understanding and involvement. One service user stated:

I will like to be involved and I will like to see them using the tool. I will like to see the smiley faces on the screen, and I will expect them to then explain to me what the results mean in terms of my risk. (Service User 15: individual interview)

Service users felt that practitioners should avoid difficult medical terms and convey information at a level appropriate to the service user:

Yes, you wouldn't want them to speak in difficult medical terms; you would want them to bring it down to the level of the person you're speaking to. (Service User 14: individual interview)

Some service users preferred being told that the tool was being used even if that increased their worry when risk information was being shared:

They probably telling you is better. A better way of using it [the tool] is that you should be told that they are using it. Even though that will still make you worry, I think that if it were something to worry it will be slightly different anyway. (Service User 1: individual interview)

Several practitioners expressed the view that patients should be told that the tool was being used to calculate risk:

If you don't tell them before using the tool it means you are not being honest. I mean you can't do anything without telling the patient, you need their consent. (Practitioner 4 [GP]: individual interview)

Practitioners admitted, ‘talking about risk is quite difficult’ (Practitioner 3 (GP): Focus Group 1), and remarked on observing patient's reactions to check, ‘do the patients actually understand me, what I am trying to tell them?’ (Practitioner 3 [GP]: Focus Group 1)*.*

In contrast, one practitioner thought they may be reluctant to inform the patient about cancer risk when they themselves were uncertain about the risk calculated or how to communicate this:

I think the only time you might do it without informing the patient is when you are uncertain, you might go back and use it and then call the patient and inform them when you are sure of the risk. (Practitioner 12 [GP]: Focus Group 3)

#### 3.2.3 Theme 3: Being open and honest

Being open and honest relates to the risk communication framework stage of treating patients with respect, which underlines the importance of recognizing the right of individuals to know the truth about their health information, as far as possible and in the interests of the individual.

Participants felt that being open and honest, including being told that a cancer risk assessment tool was being used, what the result was and what it meant for the individual patient, would help to avoid misunderstanding, whereas not telling patients the truth could affect trust between patients and practitioners. This was reflected in the following comments:

When I go to the doctor I expect to be honest with them and be clear as best as I can and you would expect the same from the practitioner, open conversation, open details from both sides to avoid misunderstanding. (Service User 19: individual interview)

Specifically, service users felt it was important to know the implications of a quantified cancer risk including whether they had a chance of not getting cancer or surviving cancer if they were diagnosed with the condition, even if this led to increased worry. A service user said, ‘I will like to be told the truth about what this 10% means and whether I've got a chance’ (Service User 3: individual interview).

Similar to the views of service users, a practitioner said:

I will be quite open and honest with them that, you've come with these symptoms, some of them are already in, and we can use the tool to work out what it is. If you bear with me I will check your risk and I could put those figures and what is coming out is your risk, and we can try that. (Practitioner 11 [GP]: Focus Group 2)

#### 3.2.4 Theme 4: Providing time for listening, explaining and reassuring in the context of a professional approach

In relation to the risk communication framework stage of explaining to patients what risk information means, the provision of time for listening, explaining and reassuring patients in a professional way was expressed by participants as important for conveying cancer risk information. Indeed, service users felt that practitioners should take time to talk to patients to gain their confidence and show they cared:

You wouldn't want to feel that you've been rushed, you would want them to take time to talk with you, and if they try to cut this conversation short you would think that they didn't care, and again that could reduce your confidence. (Service User 12: individual interview)

Practitioners also expressed the need to provide more time to provide explanations to patients:

What I feel is, I would try and give as much time as possible and be as accurate as possible. (Practitioner 1 [GP]: individual interview)

Service users appreciated that practitioners would need longer consultations:

Practitioners in general practice would need more time to use the tools in consultations. (Service User 7: individual interview)

Practitioners felt that this could be achieved by including cancer risk assessment tools in consultation software such as SystmOne, a commonly used general practice software package.

But we can target and do what we need, so unless we really suspect cancer. Also, it's time consuming but I think it will be quite quick if QCancer could be part of SystmOne. (Practitioner 11 [GP]: Focus Group 1)

## 4 DISCUSSION

### 4.1 Main findings

We found a range of ways of improving how cancer risk information could be communicated to patients during primary care consultations. These included the following: personalizing risk information arising from the use of a cancer risk assessment tool; informing and involving patients when using the tool; sharing risk information honestly; and providing sufficient time for listening, explaining and reassuring patients in the context of a professional approach. Some findings contradicted others; for example, uncertainty about risk led to reluctance to openly and honestly share information.

The views of participants in this study mapped closely to the stages of the risk communication framework. Although participants in the study had not actually used the tools in practice, their views were based on a vignette explaining how the tool would work in practice. This means that practitioners can draw on the framework and the findings of this study to aid effective communication of cancer risk in this context.

A novel outcome of this study is the use of the views of service users and practitioners to inform how to communicate with patients when using cancer risk assessment tools during general practice consultations.

### 4.2 Comparison with existing literature

This study adds to previous evidence on the importance of personalizing information to suit the educational level, cultural background and the general level of understanding of individual patients,^18^ as many patients, including those with cancer, prefer information specific to them (such as that derived from their own medical records) rather than more general information.^19^ It also supports previous evidence that use of simple visual aids can enhance doctor–patient communication,^20^ relating to the risk communication framework stage of telling patients and explaining what risk information means.^15^ Dikomitis and colleagues^21^ found problems in the design of electronic decision support tools, resulting in a recommendation for further development of these tools to make them more user friendly, and evidence from a systematic review suggested that communication tools were more likely to increase patients’ understanding if they were more structured and interactive, with illustrations such as bar charts, helping to convey understanding more than other types of graphical representation.^22^

In another systematic review investigating design features of graphs in health risk communication,^23^ there was evidence of patients being more able to recognize proportions with part‐to‐whole sequential icon arrays (ie icons arranged in an ordered pattern)^24^ than randomly arranged icon arrays,^25^ or jittered icons (ie small unsteady or difficult to visualize icons).^26^ This may explain the dislike of random‐arrangement arrays found in this study and also reported in a previous qualitative study.^27^ Participants thought that scattering blue ‘sad’ faces gave the impression that they were more numerous, that the risk perceived was higher, and that better structured graphics would ensure a visual representation which was clearer and easier for patients to understand. The importance of such design features supports the notion that the design characteristics of an intervention can help its implementation.^28^

The literature on communicating results of (asymptomatic) screening is also sparse but highlights the importance of timely and effective communication, preferably verbal and face‐to‐face (rather than by letter or face‐to‐face) on patient understanding although the findings for anxiety or worry were mixed.^29^ Providing accurate information and sharing decision making overall increased uptake of screening behaviour.^30^ The other key difference with screening asymptomatic individuals is the presumption that the Wilson–Jungner criteria^31^ have been met for the screening test, whereas there has been limited evidence presented of the outcomes of cancer risk assessment tools in terms of their acceptability, feasibility, false negatives and false positives and survival or patient experience outcomes.

Although service users in our study did not express difficulty in understanding the QCancer risk information, difficulty for some patients in understanding numbers could also complicate risk communication.^32^ Around 40% of high school graduates in one study could not perform basic numerical operations, such as converting 1% of 1000 to 10 out of 1000, presenting a major barrier to understanding or interpreting health statistics.^33^ Physicians may also find statistical information difficult to interpret and explain,^34^ and some practitioners in our study did express uncertainty about the percentage threshold at which to refer a patient.

Being open and honest when conveying cancer risk to patients was another communication strategy cited as important by service users and practitioners. That is, being open and honest has a potential to increase trust and reassurance, despite the potential to increase worry. Truthfulness relates to the risk communication framework stage of treating patients with respect,^15^ which is ethically important, contributing to building trust between patients and practitioners, as well as promoting patient autonomy and empowerment. Practitioners are required to communicate effectively, which includes delivering bad news and sharing required information in a professional and responsible manner.^35^

We found no demographic differences in the views of either service user or clinician participants on telling patients the truth about their cancer risk, although a recent study found both similarities and differences in preferences of men and women for truth‐telling and decision making.^36^ According to Chen et al, men and women had similar views of wanting to know about their medical condition, direct and frank truthfulness, and assistance in decision making for treatment. Truth‐telling differed by gender in the following ways: women wanted family members present for confirmation of diagnosis, whereas men did not; men preferred truth‐telling for only key points of their cancer, whereas women wanted detailed information; and men did not want to know their survival period, whereas women wanted this information.^36^

When using a cancer risk assessment tool, although a few service users did not mind if they were not informed and some practitioners felt they might not inform patients in some circumstances, most participants agreed that to effectively communicate cancer risk, patients should be informed and involved in the use of a cancer risk assessment tool during the consultation. This position of informing and involving patients in the use of the tool supports the notion that people need to be informed about their health‐care options.^13^ It also relates to the risk communication framework stage of making patients partners in the use of a cancer risk assessment tool.^15^

Patient involvement here refers to the participation of patients in making shared decisions about their care including referral for further cancer investigations, diagnosis and treatment.^37^, ^38^, ^39^ When sharing cancer risk information with patients, clinicians need to observe and check patients’ reactions to assess their level of understanding and the extent to which they want to be involved in making decisions.^40^ Practitioners can sometimes underestimate the degree to which patients wish to be informed about or involved in decisions about their health^41^; that is, decisions are sometimes made assuming what patients prefer,^42^ rather than involving them in the decision‐making process. If patients were not informed when a cancer risk assessment tool was being used, this could detract from gathering their views or preferences in the decision‐making process, as some patients may want to be informed about available options but may not want to be involved in the entire decision.^43^ It could also depart from the more widespread acknowledgement that people should be enabled to be involved in decisions about their care.^13^ Participants in this study, both service users and practitioners, agreed that practitioners should involve patients during consultations when using a cancer risk assessment tool.

Providing time for listening, informing, explaining and reassuring patients in the context of a professional approach was also cited by participants as important for improving effective communication. As resources and time are often limited in general practice, the emphasis here is on practitioners working within time constraints to explain risk information, as far as possible, to enhance patients’ understanding as insufficient time could impair the quality of communication. This supports the argument for extending the ten minute consultation in general practice to accommodate additional tasks such as using a cancer risk assessment tool. This relates to the risk communication framework stage of explaining to patients what the cancer risk information means,^15^ and importantly, making clear that a risk of cancer is not the same as a diagnosis of cancer. From the perspective of service users and practitioners in this study, not listening or not making efforts to explain issues to patients could adversely affect an otherwise trusted patient–practitioner relationship.

Practitioners should explain using lay terms rather than technical medical expressions which could leave patients feeling worried and anxious, not understanding what options are available, or with erroneous expectations of possible benefits and harms. A previous systematic review showed that when patients use decision aids, they improve their knowledge of the available options; are helped to have more accurate expectations of possible benefits and harms or barriers; positively affect communication with their health practitioner; and reduce time required for the consultation.^44^

The Risk Analysis Framework^15^ provided an initial framework for the analysis^16^ although some findings overlapped with more than one concept in the framework and other findings suggested that communication was more complex than the framework suggested. For example, although calculating risks correctly (‘getting the numbers right’), communicating and explaining the risks (‘telling the numbers and explaining what they mean’) were supported to a large extent by our findings, it can be seen that how cancer risk was communicated was a much more complex task. Similarly, risks of cancer may be perceived differently to other risks and the decision to act or defer action and how to do this in a way that is ethical, benefits the patient and supports their autonomy is likely to be different for each interaction depending on the perception of the patient and clinician and the relationship between them.

### 4.3 Strengths and limitations

The data collection strategy was flexible allowing the use of individual interviews (which provided information from service users and practitioners) and focus groups which facilitated the collection of diverse and detailed information from practitioner colleagues who decided to meet in a group, rather than being interviewed individually. Individual interviews provided time for clinicians to provide in‐depth information, prompted by the interviewer and topic guide, whereas the clinician focus groups benefitted from interaction and discussion between participants.

A key strength of the study was that service user and practitioner perspectives could be compared to highlight areas of agreement and disagreement between them. Another strength of the study was the achievement of data saturation in codes (ie no new ideas expressed by participants) and meaning (ie all ideas expressed were understood during the interpretation).^17^ We followed the Consolidated Criteria for Reporting Qualitative Studies^45^ (see Table S1), to ensure transparency and trustworthiness in reporting our research.

One limitation was that all nineteen service user participants were of White British ethnicity. The lack of representation from ethnic minority people is a limitation because GPs could use cancer risk tools with these patients and their views would be relevant and important. Although study publicity was circulated widely, it is possible that people from ethnic minority groups did not see the advertisement, were unable to understand it due to language difficulties, or had less interest in participating in the study. Previous research suggests that members of minority ethnic groups are less likely to participate in research studies if they lack confidence in language or understanding of the topic being researched.^46^, ^47^, ^48^ They are more likely to participate if they are approached with sensitivity and they perceive the study to be beneficial.^46^, ^49^

### 4.4 Implications for practice and research

News about cancer risk could cause undue anxiety or worry to patients. The findings from this study will help primary care practitioners to appropriately communicate cancer risk information to patients and minimize patient anxiety and worries during consultations. This paper addresses the issue of how best to communicate cancer risk information to patients presenting to primary care with symptoms which could be those of cancer. Further research on other barriers and facilitators to implementation of cancer risk assessment tools, with particular reference to QCancer, should be conducted.

## 5 CONCLUSION

Communication strategies suggested by participants in this study could be used to enhance the discourse between patients and practitioners when using cancer risk assessment tools during primary care consultations. As recognized in the Risk Analysis Framework that informed this study, the different communication strategies may be combined as appropriate to enhance communication in this context.

## CONFLICT OF INTEREST

There are no conflicts of interest for Akanuwe, Black, Owen or Siriwardena.

## AUTHOR CONTRIBUTION

Siriwardena had the original idea for the study. The study was designed by Akanuwe and Siriwardena, supported by Owen and Black. Fieldwork and analysis was conducted by Akanuwe supported by Owen, Black and Siriwardena. Akanuwe wrote the first draft of the paper, and all authors edited and approved the final paper. Siriwardena is guarantor for the paper.

## ETHICAL APPROVAL

The study was approved by the University of Lincoln School of Health and Social Care Research Ethics Committee.

## Data Availability

The anonymized qualitative datasets will be available on request through the University of Lincoln repository.
